# Quantitative Intracellular pH Determinations in Single Live Mammalian Spermatozoa Using the Ratiometric Dye SNARF-5F

**DOI:** 10.3389/fcell.2019.00366

**Published:** 2020-01-17

**Authors:** Julio C. Chávez, Alberto Darszon, Claudia L. Treviño, Takuya Nishigaki

**Affiliations:** Departamento de Genética del Desarrollo y Fisiología Molecular, Instituto de Biotecnología, Universidad Nacional Autónoma de México, Cuernavaca, Mexico

**Keywords:** intracellular pH, alkalization, spermatozoa, dual emission, image splitter, ratiometric

## Abstract

Intracellular pH (pH_*i*_) plays a crucial role in mammalian sperm physiology. However, it is a challenging task to acquire quantitative single sperm pH_*i*_ images due to their small size and beating flagella. In this study, we established a robust pH_*i*_ imaging system using the dual-emission ratiometric pH indicator, SNARF-5F. Simultaneous good signal/noise ratio fluorescence signals were obtained exciting with a green high-power LED (532 nm) and acquiring with an EM-CCD camera through an image splitter with two band-pass filters (550–600 nm, channel 1; 630–650 nm, channel 2). After *in vivo* calibration, we established an imaging system that allows determination of absolute pH_*i*_ values in spermatozoa, minimizing cell movement artifacts. Using this system, we determined that bicarbonate increases non-capacitated human pH_*i*_ with slower kinetics than in mouse spermatozoa. This difference suggests that distinct ionic transporters might be involved in the bicarbonate influx into human and mouse spermatozoa. Alternatively, pH_*i*_ regulation downstream bicarbonate influx into spermatozoa could be different between the two species.

## Introduction

The pH is fundamental for most proteins to ensure their proper function, as it influences the electrostatic status of their side chains that, in turn, affect protein structure (folding and conformation) and their interaction with other molecules (Zhou and Pang, 2018). Therefore, intracellular pH (pH_*i*_) changes serve as crucial signals in many cell types.

In spermatozoa, pH_*i*_ critically regulates motility (Ho et al., 2002; Nishigaki et al., 2014). In mammals, spermatozoa remain quiescent in the epididymis due to the acidic environment created by vacuolar-type H^+^-ATPase (V-ATPase) found in the apical plasma membrane of epithelial cells (Acott and Carr, 1984; Brown et al., 1997). Flagellar beating is suppressed in acidic environments as dynein ATPases, the motor molecules that propel the flagellum, are highly pH_*i*_ dependent (Crhisten et al., 1983). Upon ejaculation and contact with the seminal fluid sperm pH_*i*_ increases, and the flagellum starts beating. The initial flagellar beat is symmetric with low amplitude and high frequency. Subsequently in the oviduct, the flagellar beat pattern becomes vigorous (asymmetric with high amplitude and low frequency), a process called hyperactivation (Ho and Suarez, 2001). Hyperactivated motility is essential for mammalian spermatozoa since it is required to approach the oocyte and to penetrate its investments (Stauss et al., 1995; Suarez and Pacey, 2006). In order to induce and maintain hyperactivation, an increase in intracellular Ca^2+^ concentration ([Ca^2+^]_i_) is required (Ho et al., 2002), which is mediated through a sperm-specific Ca^2+^ channel, named CatSper (Ren et al., 2001). Although there are species-specific activation mechanisms of CatSper (Lishko et al., 2011), this channel is moderately voltage dependent and highly up regulated by intracellular alkalization (Kirichok et al., 2006). In mouse, the sperm-specific Na^+^/H^+^ exchanger (sNHE) is essential for the regulation of sperm motility and has been proposed as an activator of CatSper by elevating pH_*i*_ (Wang et al., 2003; Navarro et al., 2008). On the other hand, in human spermatozoa, a voltage-gated H^+^ channel (Hv1) has been documented to be the main H^+^ transporter that activates CatSper rather than sNHE (Lishko et al., 2010). In sea urchin sperm, CatSper is a predominant player in chemotaxis toward sperm-attracting peptides (Seifert et al., 2015; Espinal-Enríquez et al., 2017) and sNHE has been shown to be critical for modulating CatSper activity (González-Cota et al., 2015; Windler et al., 2018).

External bicarbonate (${\text{HCO}}_{3}^{-}$) is fundamental for capacitation in mammalian spermatozoa (Lee and Storey, 1986; Visconti et al., 1995). Both the pH and the ${\text{HCO}}_{3}^{-}$ concentration of the oviductal fluid are higher in uterine and tubal fluids compared to plasma (Vishwakarma, 1962). Moreover, pH in the rhesus monkey female tract elevates dramatically, concomitantly with ovulation (Maas et al., 1977), which might promote sperm capacitation *in vivo*. In mammalian spermatozoa, several ${\text{HCO}}_{3}^{-}$ transporters were reported as candidates to mediate ${\text{HCO}}_{3}^{-}$ influx across the plasma membrane such as Na^+^/${\text{HCO}}_{3}^{-}$ cotransporter (NBC) (Demarco et al., 2003), Cl^–^/${\text{HCO}}_{3}^{-}$ exchangers (Chavez et al., 2012), and CFTR (Hernández-González et al., 2007; Xu et al., 2007), as well as its indirect entrance via CO_2_ diffusion with subsequent hydration by intracellular carbonic anhydrases (CA) (Wandernoth et al., 2010; José et al., 2015). Besides an increase in the pH_*i*_, a cytosolic ${\text{HCO}}_{3}^{-}$ elevation is crucial for activation of the sperm soluble adenylyl cyclase (Okamura et al., 1985; Buck et al., 1999).

To understand how sperm pH_*i*_ is regulated, it is indispensable to determine where and when it changes in individual cells. Although sperm pH_*i*_ measurements in suspension have been performed using fluorescence indicators for more than three decades (Schackmann and Boon Chock, 1986; Darszon et al., 2004; Hamzeh et al., 2019), there are few reports of imaging single sperm pH_*i*_ (Zeng et al., 1996; Chávez et al., 2014, 2018; González-Cota et al., 2015). All these experiments were performed with BCECF (Rink et al., 1982), the most popular fluorescent pH_*i*_ indicator in cell physiology. This fluorescence probe is ratiometric but requires dual-excitation (Rink et al., 1982). Consequently, there is a time lag between two subsequent images excited by two different wavelengths and therefore, cell movement artifacts can be significant. Furthermore, BCECF is highly phototoxic to cells (Nishigaki et al., 2006), which was also confirmed in this study.

To overcome the BCECF disadvantages stated above we employed SNARF-5F acetoxymethyl ester (AM) (Liu et al., 2001) whose fluorescence spectra changes (shift of the peak wavelength) depending on pH (pKa: 7.2). This dye allowed us to perform dual-emission ratiometric pH_*i*_ imaging using an image splitter with a single EMCCD camera. In this report, we detail our pH_*i*_ imaging setup and conditions. Furthermore, we found kinetic differences in the pH_*i*_ changes induced by ${\text{HCO}}_{3}^{-}$ in human and mouse spermatozoa which could suggest that ${\text{HCO}}_{3}^{-}$ influx pathways are distinct in human and mouse spermatozoa.

## Materials and Methods

### Materials

Dimethyl sulfoxide (DMSO, cat. D2650), ammonium chloride (NH_4_Cl, cat. A9434), nigericin (cat. N7143), progesterone (cat. P8783), and concanavalin A (cat. C2010) were purchased from Sigma–Aldrich. Pluronic F-127 (cat. P6867), SNARF-5F AM (5-(and-6)-carboxylic acid, acetoxymethyl ester) (cat. S23923), and 2′, 7′-Bis-(2-carboxyethyl)-5-(and-6)-carboxyfluorescein, acetoxymethyl ester (BCECF AM) (cat. B1170) were obtained from ThermoFisher Scientific.

### Biological Sample Collection

#### Human Spermatozoa

Human spermatozoa samples were obtained from healthy donors under written informed consent and with the approval of the Bioethics Committee of the Instituto de Biotecnología, Universidad Nacional Autónoma de México (IBt-UNAM). Only ejaculates that fulfilled the World Health Organization guidelines were used in all the experiments (Cao et al., 2010). Motile cells were recovered using the swim-up technique in HTF medium (in mM: 90 NaCl, 4.7 KCl, 1.6 CaCl_2_, 0.3 KH_2_PO_4_, 1.2 MgSO_4_, 2.8 glucose, 0.3 pyruvic acid, 23.8 HEPES, and 21.4 lactic acid, 25 NaHCO_3_) pH 7.4 (Mata-Martínez et al., 2013). Briefly, 400 μl of liquefied semen was placed in glass test tubes and 1 ml HTF medium was carefully added on the top of the semen without mixing the phases. Samples were incubated for 1 h at 37°C under 5% CO_2_. The upper layer (700 μl) with motile spermatozoa was then collected. Cell density was determined using a Makler chamber and adjusted to 10 × 10^6^ spermatozoa/ml.

#### Mouse Spermatozoa

All experimental protocols were approved by the Bioethics Committee of the IBt-UNAM). Motile spermatozoa were obtained from epididymal cauda of 3-month-old CD-1 mouse by placing incised epididymis in an Eppendorf tube containing 1 ml of in TYH medium (in mM: 119 NaCl, 4.7 KCl, 1.7 CaCl_2_, 1.2 KH_2_PO_4_, 1.2 MgSO_4_, 5.6 dextrose, 0.5 pyruvic acid, and 20 HEPES) pH 7.4. Spermatozoa were allowed to swim-out during 15 min at 37°C. The upper layer (800 μl) with motile spermatozoa was collected and the cell density was adjusted to 10 × 10^6^ spermatozoa/ml using a Makler counting chamber (Irvine Scientific, Santa Ana, CA, United States).

### *In vitro* Fluorescence Spectra of SNARF-5F

**FIGURE 1:**
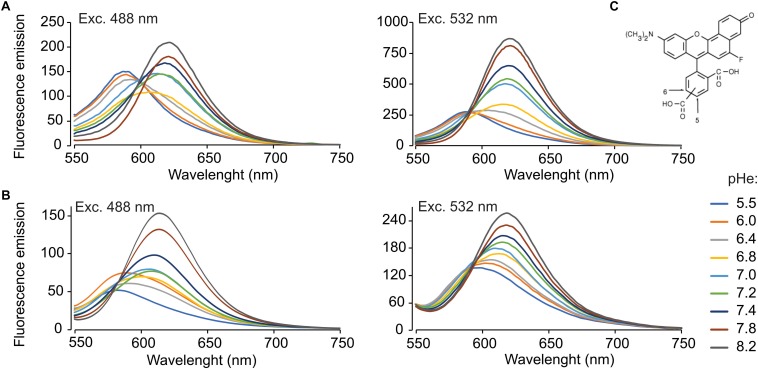
SNARF-5F emission spectra at 488 and 532 nm excitation wavelengths, in 50 mM potassium phosphate buffers at various pH values. Emission spectra of non-permeable SNARF-5F **(A)** using a fixed excitation wavelength of 488 nm (**A**,left) and 532 nm (**A**,right). Emission spectra of SNARF-5F-AM **(B)** with 10 × 10^6^ sperm/ml treated with 0.1% Triton X-100, using a fixed excitation wavelength of 488 nm (**B**,left) and 532 nm (**B**,right). The lines are representative fluorescence spectra at different pH_e_ (5.5, 6.0, 6.4, 6.8, 7.0, 7.2, 7.4, 7.8, and 8.2), indicated by color lines. **(C)** SNARF-5F chemical structure. Typically, SNARF-5F possess two emission wavelengths, at 575 and 640 nm; *n* = 4.

Fluorescence spectra of SNARF-5F were determined with a Perkin-Elmer LS 55 (Perkin-Elmer, Waltham, MA, United States) fluorescence spectrometer using the software FL WinLab version 4.00.03 for data acquisition (Figure 1). SNARF-5F non-permeable and AM versions were used at 20 μM final concentration in 50 mM potassium phosphate buffer (see table in Supplementary Figure S1). Multiple spectra were acquired using various excitation wavelengths (405, 440, 465, 488, 510, 532, and 543 nm) and different pH_e_ (5.5, 6.0, 6.4, 6.8, 7.0, 7.2, 7.4, 7.8, and 8.2) (Supplementary Figure S1).

### SNARF-5F and BCECF Incorporation Into Spermatozoa

Motile mouse/human spermatozoa (10 × 10^6/^ml) were incubated with 20 μM SNARF-5F AM in the presence of 0.1% pluronic F-127 during 90 min at 37°C with 5% CO_2_ in the dark. The cells were washed once by centrifugation at 200 × *g* for 5 min and resuspended with fresh medium. To obtain fluorescence spectra of SNARF-5F incorporated into spermatozoa, human spermatozoa loaded with SNARF-5F AM were diluted in the media of different pH_e_ as described above and treated with 0.1% Triton-X 100 detergent. Fluorescence spectra of the lysed spermatozoa were acquired at excitation wavelengths 488 and 532 nm. For single cell recordings, spermatozoa were attached to Concanavalin A-treated coverslips for 2–3 min and mounted in recording chambers.

For BCECF experiments, motile mouse/human spermatozoa (10 × 10^6/^ml) were incubated with 1 μM BCECF AM without pluronic acid during 15 min at 37°C with 5% CO_2_ in the dark and the unincorporated dye was removed by centrifugation as the case of SNARF-5F.

### Imaging Setup

**FIGURE 2:**
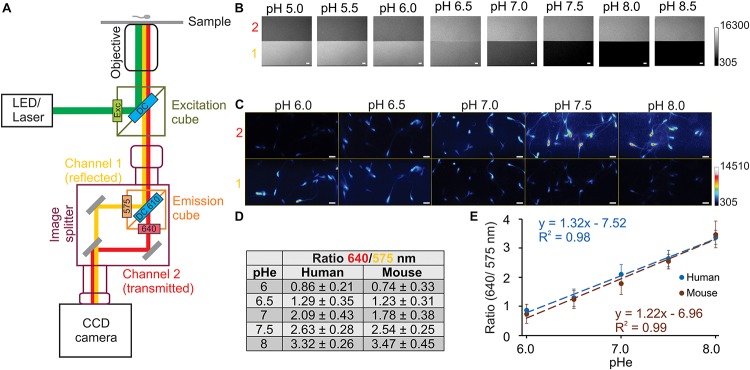
Imaging set-up configuration to visualize the dual emission wavelengths of SNARF-5F. Diagram of imaging set-up **(A)**, indicating LED (532 nm) or laser (488 nm) as an illumination source. A different excitation cube (green square) was used for the LED set-up (ET 530/30X, dichroic mirror DC 550 LP) and the laser set-up (D485/25X, dichroic DC510 LP). To visualize at the same time the two emission wavelengths of SNARF-5F, we used an image splitter (in purple), which has an emission cube (orange square) (ET575/50M, ET640/20M, and dichroic mirror DC610 LP), that divides the emission in reflected (channel 1, corresponding to emission wavelength 575 nm) or transmitted (channel 2, corresponding to emission wavelength 640 nm) images. The image splitter is coupled to the microscope on one side, and to the detector (CCD camera) on the other. Fluorescence images obtained from epifluorescence (laser set-up) microscope with 60× (Plan Apo N, 1.49 numerical aperture) objective, using 20 μM non-permeable SNARF-5F **(B)** or 20 μM SNARF-5F-AM loaded in human and mouse (not shown) spermatozoa in the presence of nigericin 10 μM **(C)** in 50 mM potassium phosphate buffers at indicated pH. **(D)** Ratio values were obtained (referred from panel **C**) at each pH_e_ (6.0, 6.5, 7.0, 7.5, and 8.0) in human and mouse spermatozoa, using channel 1 (575 nm) and channel 2 (640 nm) fluorescence values. **(E)** Lineal correlation between pH_e_ and fluorescence ratio in human and mouse spermatozoa, obtaining *R*^2^ = 0.99 and 0.98, respectively. Numbers 1 (yellow) and 2 (red) to the left in panels **B** and **C** refer to channel 1 (emission 575 nm) and channel 2 (emission 640 nm). Scale bar in panels **B** and **C** is equal to 10 μm; *n* = 3.

Single cell images were acquired using two different setups: (1) Olympus iX71 LED-light source epifluorescence microscope and (2) Olympus iX81 laser widefield/total internal reflection fluorescence (TIRF) microscope (Olympus, Japan). The LED setup was equipped with a PlanApo N 60X/1.42 oil objective and a 3A 532 nm LED coupled to Opto-LED light controller (Cairn Research, United Kingdom). The laser setup was equipped with an Apo N (TIRF) 60X/1.49 oil objective and a 488 nm laser with high speed imaging shutter. To acquire dual emission images of SNARF-5F, an image splitter OptoSplit II (Cairn Research, United Kingdom) was used for both setups, LED (with band-pass filter ET 530/30X) and laser. To acquire the images with SNARF-5F, a wide band-pass filter ET 575/50 M (channel 1) and a band-pass filter ET 640/20 M (channel 2) were employed as dual emission filters combined with a dichroic mirror DC 610lp (Chroma Technology, United States) (Figure 2). For BCECF experiments, a 3.15 A 465 nm LED (Luminus Devices, Woburn, MA, United States) with bandpass filter HQ 480/40X was used for excitation light, combined with a dichroic mirror (Q505lp) and an emission filter (HQ 535/50M) (Chroma Technology, United States). Each setup has a 512 × 512 Andor iXon 3 EMCCD camera (model X3 DU897E-CS0) (Oxford Instruments, United Kingdom).

Images were acquired with the software Andor iQ version 2.9.1 (LED set-up) (Oxford Instruments, United Kingdom) and Xcellence version 1.2 (laser set-up) (Olympus, Japan). Fluorescence images of both SNARF-5F and BCECF were taken with 1 × 1 binning, 5 images/s (5 ips), with an exposure time of 10 ms for the LED setup and 30 ms (laser potency 35%) for the laser setup. Images were analyzed with ImageJ version 1.52n (NIH, United States), obtaining mean fluorescence intensities, selecting heads and flagellum as regions of interest.

### *In vivo* pH_*i*_ Calibration

To convert fluorescence data to pH values *in vitro*, the following equation is commonly used:

$$\text{pH}={\text{pK}}_{\text{a}}-\log\left[\frac{R-R_{\text{B}}}{R_{\text{A}}-R}\times \frac{F_{\text{B}\,\left({\text{λ}}_{2}\right)}}{F_{\text{A}\,\left({\text{λ}}_{2}\right)}}\right]$$

where *R* is the ratio *F*λ_1_/*F*λ_2_ of fluorescence intensities (*F*) measured at two wavelengths λ_1_ and λ_2_ and the subscripts A and B represent the values at the acidic and basic conditions, respectively (Whitaker et al., 1991). However, it is difficult to maintain live spermatozoa in highly acidic and alkaline condition to obtain *F*_B(λ_2_)_/*F*_A(λ2)_ values from the same cells. Therefore, we performed *in vivo* pH_*i*_ calibration by fixing external pH (pH_e_) between 6.0 and 8.0 as reported previously (Grillo-Hill et al., 2014). Briefly, spermatozoa suspensions were incubated for 15 min with calibration medium (in mM: 120 KCl, 25 HEPES, 1 MgCl_2_, and 0.01 nigericin, at different pH_e_: 6.0, 6.5, 7.0, 7.5, and 8.0, adjusted with KOH). We measured the fluorescence intensity at two emissions, 575 (channel 1) and 640 nm (channel 2), always subtracting the fluorescence background value in each channel. As fluorescence ratio values (*R*_*F*__640__/F__575_) have a lineal relation with pH_*i*_ values (between 6.0 and 8.0) (Figures 2D,E), we used the following equations to estimate pH_*i*_ from the fluorescence ratio values, for human: pH_*i*_ = (*R*_*F*__640__/F__575_ + 6.96)/1.22 and for mouse: pH_*i*_ = (*R*_*F*__640__/F__575_ + 7.52)/1.32.

### Statistical Analysis

The results are expressed as the mean ± SEM of at least three independent experiments (three different donors or mice), with a minimum of 200 cells per condition. The data were analyzed by a comparison test between the groups, using the non-parametric Mann–Whitney *U*-test with 95% statistical significance. The paired tests were carried out comparing the head and the flagellum of the same cell. Additionally, the Bonferroni correction was used when multiple comparisons were made.

## Results

### Emission Spectra of SNARF-5F With Distinct Excitation Wavelengths

To perform ratiometric fluorescence measurements with a good signal to noise ratio (S/N ratio), it is important to acquire bright fluorescence images in both channels. In other words, if we detect dim fluorescence signals in one channel, the S/N ratio of the dual-emission ratio values become undesirably low even when we detect bright signals in the other channel. As spermatozoa possess a quite reduced cytoplasm, it is crucial to use an appropriate excitation wavelength and emission filters. Therefore, we first determined the fluorescence spectra of SNARF-5F at several pH_e_ values (5.5–8.2), exciting with various wavelengths (405–543 nm). As shown in Supplementary Figure S1, the longer excitation wavelength (longer than 465 nm) gives the higher fluorescence intensities in all emission wavelengths we explored (550–750 nm). Namely, 543 nm produced the highest fluorescence values.

Figure 1 illustrates the fluorescence spectra of SNARF-5F at different pH_e_ (5.5–8.2) excited at 488 and 532 nm. At both exciting wavelengths, the fluorescence intensities around 575 nm (the first peak) decrease when the pH increases, while those of around 640 nm (the second peak) increase at the same conditions. When exciting at 532 nm, the relative fluorescence intensities within the first peak at different pH_e_ are much smaller than those within the second peak, as reported in the original article of SNARF-5F (Liu et al., 2001). Conversely, the relative fluorescence intensities within the two peaks became almost equal when 488 nm was used as excitation light (Figure 1A). In spite of this favorable feature, their absolute fluorescence intensities are small. Considering these characteristics, we selected 532 nm as the best compromise between brightness and peak balance for this study.

To evaluate the incorporation of the membrane permeant dye SNARF-5F AM into the spermatozoa, we incubated human spermatozoa with 20 μM of this dye for 90 min. After the excess dye was washed out by centrifugation, the cells were lysed with 0.1% Triton X-100 and the fluorescence spectra were acquired (Figure 1B). The spectra of the dye incorporated into human spermatozoa were not identical to SNARF-5F *in vitro*, suggesting that SNARF-5F AM was not completely hydrolyzed in the cell and/or some of the dye was bound to certain molecules of the cell. Nevertheless, SNARF-5F AM incorporated into the cell responded to pH_e_ changes similarly to SNARF-5F AM.

### Dual-Emission pH_*i*_ Imaging System and *in vivo* Calibration of pH_*i*_

A conventional dual-emission fluorescence imaging setup usually is composed of an epi-fluorescence microscope, a CCD camera, and a filter wheel, which exchanges two emission filters alternatively. In this type of setup, there is always a time lag between the image in one channel and the image in the other channel. Since spermatozoa are small and motile cells, the presence of a time lag between two images of each channel is undesirable. Therefore, we used an image splitter (Kinosita et al., 1991) that allows the simultaneous capture of the images from the two channels with a single camera (Figure 2A). In a common configuration of dual-emission ratiometric imaging, emission lights are divided into two components (two channels) at the isosbestic point, around 595 nm in the case of SNARF-5F AM excited by 532 nm. However, because the fluorescence intensity of the first peak (575 nm) is lower than the second peak (640 nm) as described previously (Figure 1), we separated the emission light at 610 nm (about 15 nm longer than the isosbestic point) by a dichroic mirror. Consequently, we collected a wide range of wavelengths, 550–600 nm, as the fluorescence signals in the first channel (channel 1). Then, we collected 630–650 nm wavelengths as the longer wavelength fluorescence (channel 2). This configuration gives us comparable fluorescence intensities from the two channels without the insertion of a neutral density filter (Figure 2A).

Figure 2B shows fluorescence images (gray scale) of SNARF-5F in media at different pHs, clearly demonstrating the opposite changes of fluorescence intensity between Channel 1 and Channel 2. Since fluorescence spectra of SNARF-5F and SNARF-5F AM incorporated into human spermatozoa show a slight difference (Figures 1A,B), we performed *in vivo* calibration using human spermatozoa to convert the ratio fluorescence values into pH_*i*_ values. To perform *in vivo* calibration, spermatozoa pH_*i*_ was equilibrated to the pH_e_ using high K^+^ (120 mM) media in the presence of 10 μM nigericin (an ionophore that facilitates K^+^/H^+^ exchange across the lipid bilayer). Figure 2C shows fluorescence images (pseudo color) of human spermatozoa, whose pH_*i*_ was fixed at different pH_e_ (6.0–8.0).

**FIGURE 3:**
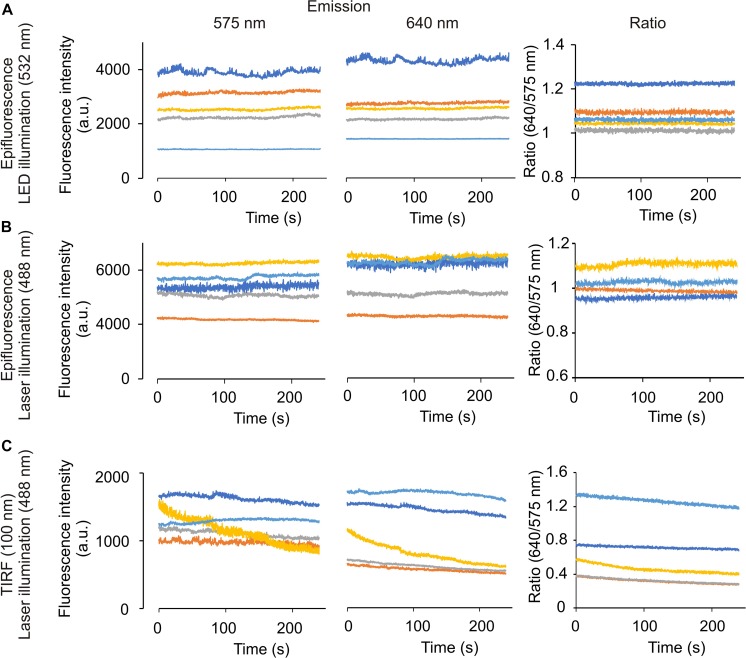
Time-lapse experiments in the microscope set-up did not cause significant photobleaching in mammal spermatozoa, using LED or laser as an illumination source. Representative recordings from emission wavelength time lapse experiments, using 20 μM SNARF-5F-AM in human spermatozoa. Images were taken every 200 ms, exposure time 4 ms with 60× objective. An image splitter was used for the experiments, allowing us to measure emission fluorescence at 575 (left) and 640 (center) nm, and obtaining the ratio from both wavelengths (right). The illumination source was LED 532 **(A)** or laser 488 nm **(B,C)**, in the epifluorescence **(A,B)** or TIRF 100 nm **(C)** configuration. Traces in each panel show representative single cell pH_*i*_. Same color at both emission wavelengths indicates the same cell; *n* = 3.

The mean ratio values of fluorescence intensities of the two channels (F640/F575) in distinct pH_*i*_ are summarized in Figure 3C and these ratio values are plotted as a function of pH_*i*_ (Figure 2D). The ratio values increase proportionally to pH_*i*_ between 6.0 and 8.0 with excellent linearity (human spermatozoa: *R*_*F*__640__/F__575_ = 1.22 × pH_*i*_ – 6.96, *R*^2^ = 0.99; mouse spermatozoa: *R*_*F*__640__/F__575_ = 1.32 × pH_*i*_ – 7.52, *R*^2^ = 0.98) (Figure 2E).

### Phototoxicity of SNARF-5F to Spermatozoa

BCECF is known to be quite phototoxic to spermatozoa and this effect can be easily detected as flagellar beat attenuation and as a decrease in the fluorescence intensity (photo-bleaching) during the intense exposure of excitation light (Nishigaki et al., 2006; González-Cota et al., 2015). In this study, we confirmed the phototoxic effect of BCECF on sperm using the same setup utilized for SNARF-5F (Supplementary Figure S2). Particularly, the 488 nm laser excitation attenuated the flagellar beat of human and mouse spermatozoa after around 60 and 20 s illuminations, respectively. Subsequently, notable photobleaching of BCECF was observed in both human and mouse spermatozoa (Supplementary Figures S2A,C). On the other hand, LED illumination caused less photobleaching in human spermatozoa (Supplementary Figure S2B), but certain level of photobleaching was still observed in 40% of mouse spermatozoa (Supplementary Figure S2D). This result suggests that mouse sperm are more susceptible to oxidative stress than human spermatozoa.

In contrast, SNARF-5F incorporated into spermatozoa is much less toxic to the cells than BCECF (Figure 3). The fluorescence intensities excited by LED and 488 nm laser (epi-fluorescence mode) did not cause photo-bleaching of the dye during our experimental periods (5 ips for 250 s) (Figures 3A,B). However, we observed a slight photobleaching of SNARF-5F when excited by the 488 nm laser in the TIRF configuration (Figure 3C). This photobleaching was negligible when we reduced the frequency of image acquisition from 5 to 2.5 ips (data not shown).

### Comparison of Epi-Fluorescence and TIRF Images

**FIGURE 4:**
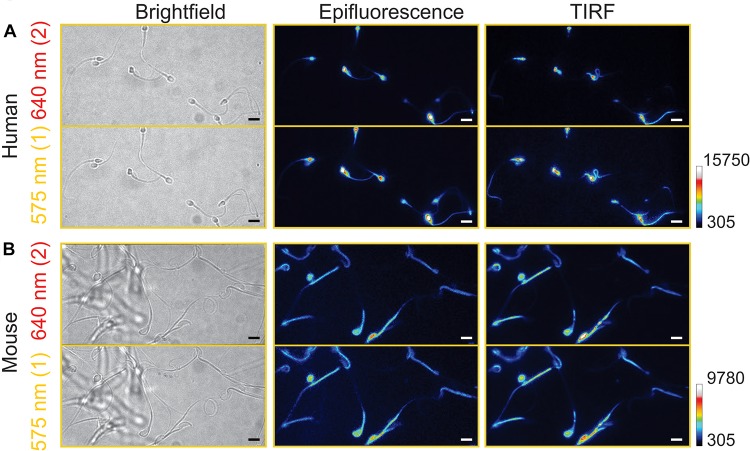
Comparison between epifluorescence and total internal reflection fluorescence (TIRF) images. Representative images from human **(A)** and mouse **(B)** spermatozoa in the two emission channels for SNARF-5F dye, 640 (red) and 575 nm (yellow). Images were taken using the epifluorescence (center) or TIRF (right) configuration. For reference, brightfield images (left) are shown. Scale bar is equal to 10 μm. Reference bar for fluorescence intensity is also depicted. Scale bar is equal to 10 μm; *n* = 3.

In our previous study of pH_*i*_ imaging (epi-fluorescence mode) of sea urchin spermatozoa using BCECF, fluorescence intensities of the flagellum were much lower than those of the heads and their fluorescence signals were noisy with a poor S/N ratio (González-Cota et al., 2015). We thought that the use of TIRF would improve this aspect, avoiding the saturation of fluorescence signal in the head. However, the difference of SNARF-5F images between the two configurations (epi-fluorescence and TIRF) was relatively small in both human (Figure 4A) and mouse spermatozoa (Figure 4B). Particularly, in mouse spermatozoa, the TIRF fluorescence signals in the head and the flagellum (primarily mid piece) are very similar to the epi-fluorescence images. This result is probably due to the thin hook-like shape of the mouse sperm head. As a consequence, an important difference between the two systems (epi-fluorescence and TIRF) is not significant for mouse spermatozoa.

### Spermatozoa Responses to pH_*i*_ Manipulation

**FIGURE 5:**
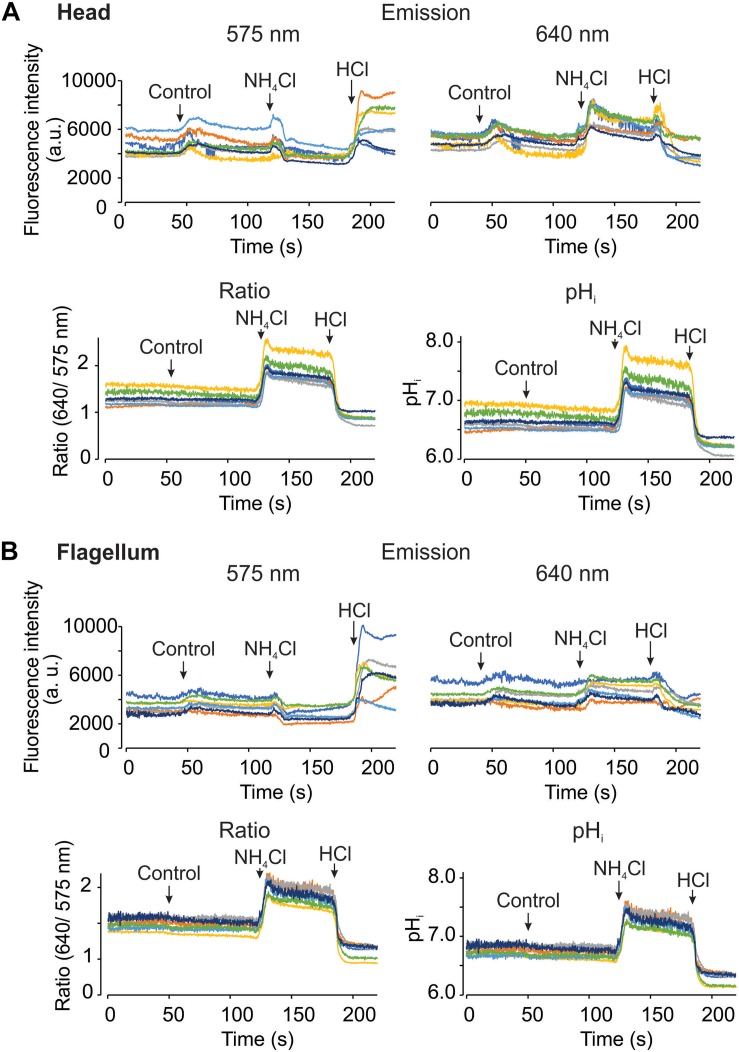
It is possible to measure pH_*i*_ using SNARF-5F in human spermatozoa, in the head and flagellum regions. Representative recordings from head **(A)** and flagellum **(B)** regions, measuring fluorescence changes at the two emission wavelengths of SNARF-5F, 575 (Top left) and 640 nm (Top right). Ratio recordings (Bottom left) are obtained from both emission fluorescence values, and converted to pHi (Bottom right) utilizing the calibration curve as shown in Figure 2. The micropipette manual addition of HTF medium (control), 10 mM NH_4_Cl, and 5 mM HCl is indicated by arrows in each panel. Traces in each panel show representative single cell pH_*i*_ responses. Same color at both emission wavelengths and in both regions (head and flagellum) correspond to the same cell; *n* = 3.

**FIGURE 6:**
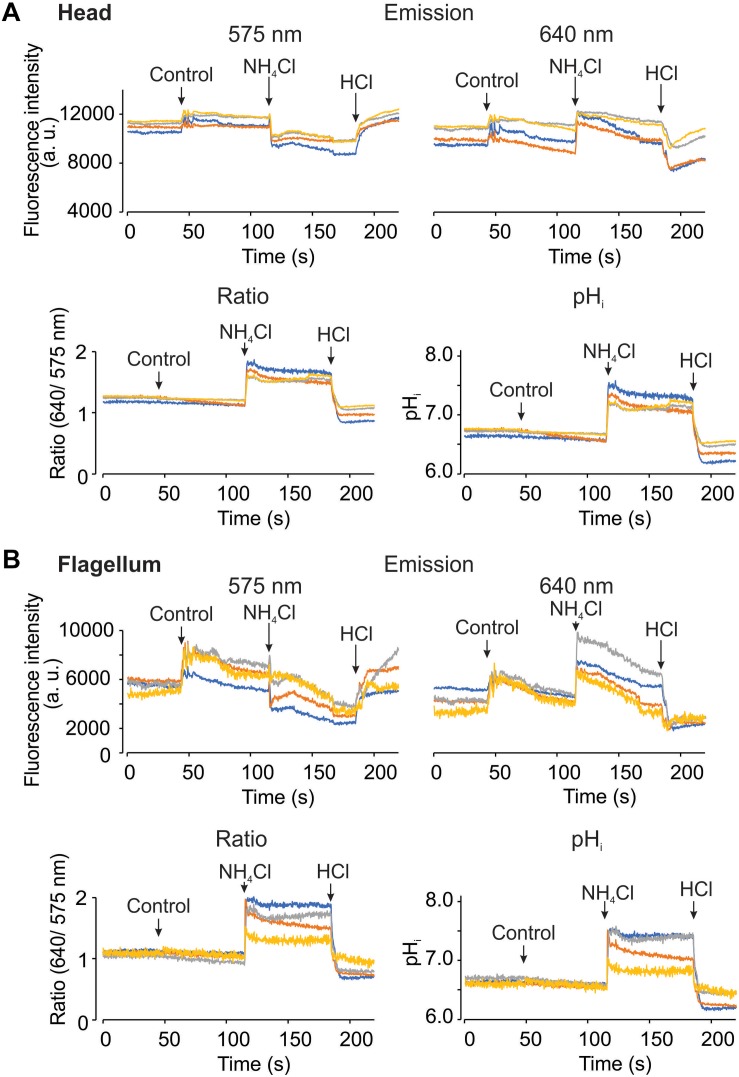
It is possible to measure pH_*i*_ using SNARF-5F in mouse spermatozoa, in the head and flagellum regions. Representative fluorescence recordings from head **(A)** and flagellum **(B)** regions, measuring changes at the dual emission wavelengths of SNARF-5F, 575 (Top left) and 640 nm (Top right). Ratio recordings (Bottom left) are obtained from both emission fluorescence values, and converted to pH_*i*_ (Bottom right) using the calibration curve shown before. The micropipette manual addition of TYH medium (control), 10 mM NH_4_Cl, and 5 mM HCl are indicated by arrows in each panel. Traces in each panel show representative single cell pH_*i*_. The same color at both emission wavelengths and in both regions (head and flagellum) correspond to the same cell; *n* = 3.

Using the epi-fluorescence configuration with the LED as a light source, we acquired fluorescence images upon pH_*i*_ manipulations. During image acquisition, we added HTF or TYH medium as control in human and mouse spermatozoa, respectively. As additional control, 10 mM NH_4_Cl and 5 mM HCl were added to increase and reduce the pH_*i*_, respectively. The upper panels of Figures 5A,B show human sperm fluorescence signals from the two channels in the head and the flagellum, respectively. Changes of fluorescence intensities were observed during the additions even in control conditions (indicated with arrows). Also, fluorescence signals from some cells are noisy probably due to the continuous movement associated to the flagellar beat. Once the dual emission signals were converted into the ratio and pH_*i*_ values (Figures 5A,B, lower panels), the problems of addition artifacts and movement were eliminated in the both regions, demonstrating the advantage of the dual-emission ratiometric imaging. Additionally, the effects of NH_4_Cl and HCl can be clearly observed as an increase and a decrease of the ratio and the pH_*i*_, respectively. Figure 6 basically demonstrates the same results as Figure 5 but using mouse spermatozoa. In this experiment, the fluorescence signal of the flagellum arises mainly from the mid piece since mouse spermatozoa flagellum is much longer than that of human spermatozoa and therefore it is difficult to capture the image of the entire flagellum of mouse spermatozoa. In these experiments, the average pH_*i*_ of non-capacitated human and mouse spermatozoa was 6.72 ± 0.19 (SEM) and 6.63 ± 0.23 (SEM), respectively; *n* = 3.

### Response to ${\text{HCO}}_{3}^{-}$

**FIGURE 7:**
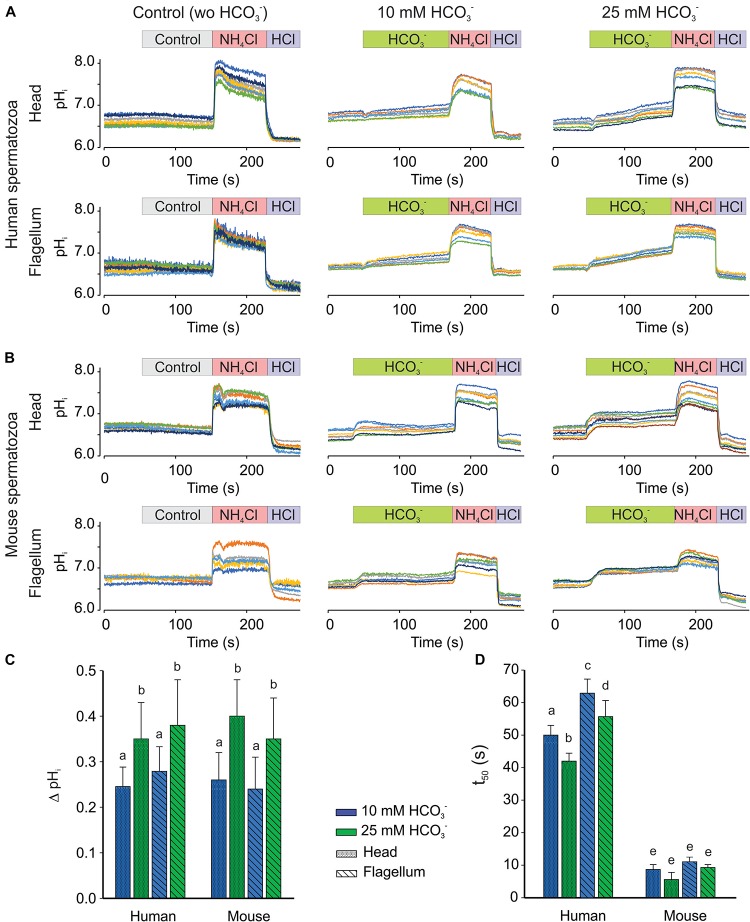
${\text{HCO}}_{3}^{-}$ increased pH_*i*_ in a concentration-dependent manner in both, head and flagellum, regions using human and mouse spermatozoa. Representative recordings from human **(A)** and mouse **(B)** spermatozoa, measuring pH_*i*_ using 20 μM SNARF-5F in head (Top) and flagellum (Bottom) regions. The perfused addition of medium (HTF and TYH for human and mouse, respectively) (left, control in gray rectangle), 10 (center in green rectangle) or 25 mM (right in green rectangle) ${\text{HCO}}_{3}^{-}$ are showed. As positive controls, perfused addition of 10 mM NH_4_Cl (red rectangle) and 5 mM HCl (purple rectangle) are showed in each panel. Traces in each panel show representative single cell pH_*i*_. Same color in both, head and flagellum, indicate to the same cell. Maximum change in pH_*i*_ (ΔpH_*i*_) **(C)** and average of *t*_50_ **(D)**, time to reach 50% of the maximum fluorescent intensity, before and after 10 (blue bars), 25 (green bars) mM ${\text{HCO}}_{3}^{-}$ addition, in head (shaded) or flagellum (diagonal lines) regions. The bars in **C,D** indicated means ± SEM. Different letters indicate significant differences at the *p* ≤ 0.05 level, according to Mann–Whitney *U*-test; *n* = 5.

To obtain new insights of mammalian spermatozoa pH_*i*_ regulation, we determined the effect of ${\text{HCO}}_{3}^{-}$ (10 and 25 mM) on pH_*i*_ in non-capacitated human and mouse spermatozoa using our dual-emission imaging system (Figure 7). In these experiments, we confirmed that ${\text{HCO}}_{3}^{-}$ increases the pH_*i*_ of human (Figure 7A) and mouse (Figure 7B) spermatozoa, in a concentration-dependent manner. We did not observe statistical differences in the pH_*i*_ increase induced by ${\text{HCO}}_{3}^{-}$ between human and mouse spermatozoa (Figure 7C). However, we found a significant difference in the kinetics of the pH_*i*_ increase between the two species. Namely, ${\text{HCO}}_{3}^{-}$ rapidly increases the pH_*i*_ of mouse spermatozoa, and the time to reach 50% of the maximum pH_*i*_ increase (*t*_50_) was around 10 s. In contrast, ${\text{HCO}}_{3}^{-}$ increases human sperm pH_*i*_ gradually with a longer *t*_50_ (40 s) in our experimental conditions (Figure 7D). Moreover, the pH_*i*_ increase in human spermatozoa was slightly, but significantly, slower in the flagellum compared to the head with 10 and 25 mM ${\text{HCO}}_{3}^{-}$ additions.

### Response to Progesterone in Human Spermatozoa

**FIGURE 8:**
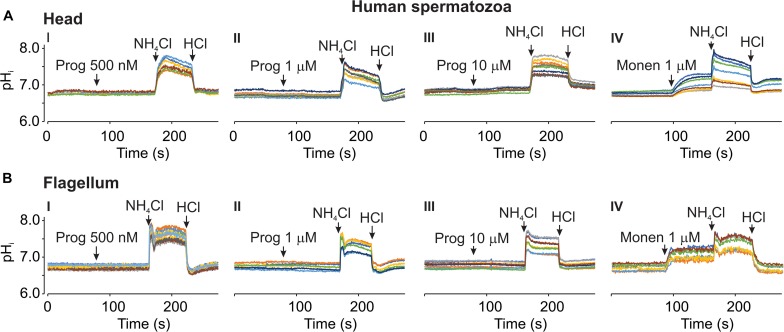
Progesterone did not changed pH_*i*_ in human spermatozoa. Representative recordings using 20 μM SNARF-5F in human spermatozoa in the head **(A)** and flagellum **(B)** regions. Micropipette manual progesterone (Prog) additions of 500 nM (I), 1 μM (II), and 10 μM (III), as well as 1 μM of monensin (Monen) (IV) are indicated by arrows in each panel. As controls, 10 mM NH_4_Cl and 5 mM HCl additions were performed (see arrows). Traces in each panel show representative single cell pH_*i*_. The same color at both emission wavelengths and in both regions (head and flagellum) corresponds to the same cell; *n* = 3.

In the literature, there is some controversy about the effect of progesterone on human sperm pH_*i*_. A decrease (Garcia and Meizel, 1996; Cross and Razy-Faulkner, 1997), no change (Fraire-Zamora and González-Martínez, 2004) or a slow increase (Hamamah et al., 1996) in pH_*i*_ have been reported by different groups in response to this hormone. Therefore, we determined the effect of different progesterone concentrations on pH_*i*_ in human spermatozoa. Figure 8 shows that progesterone at 500 nM (I), 1 μM (II), and 10 μM (III) did not change pH_*i*_ neither in the head (Figure 8A) nor in the flagellum (Figure 8B) of these cells. As a control we tested 1 μM monensin, a Na^+^ ionophore that exchanges Na^+^/H^+^ (Babcock, 1983). As anticipated, this ionophore alkalized pH_*i*_ in these cells in both head (Figure 8A, IV) and flagellum (Figure 8B, IV).

## Discussion

### Advantages of the New System to Determine Spermatozoa pH_*i*_

In this study we established a dual-emission ratiometric imaging system using SNARF-5F AM, which has negligible photo-toxicity compared to BCECF (Figure 3 and Supplementary Figure S2). Our system allows determining mammalian spermatozoa (head and flagellum) pH_*i*_ with minimum artifacts associated to cell movements and focus alteration upon addition or exchange of bath solutions (Figures 5–7). Commonly, the ratio of dual fluorescence signals utilizes the dye isosbestic point (595 nm in our condition). However, the first peak fluorescence intensity (575 nm) of SNARF-5F excited at 532 nm is much smaller than the second peak (640 nm). Therefore, we divided the fluorescence at 610 nm, 15 nm longer than the isosbestic point, and employed a wide band-pass filter (550–600 nm) for Channel 1. In this configuration, fluorescence signals of the two channels are comparable (Figure 2), which is a critical point to obtain the ratio values with a good S/N ratio. This type of optical filter configuration (division of fluorescence signals not at the isosbestic point) could be applied to other dual-emission indicators such as GEM-GECO (Zhao et al., 2011) and Asante Calcium Red (Hyrc et al., 2013) because their dual-emission signals are quite asymmetric.

### pH_*i*_ Calibration

We observed a slight difference between the fluorescence spectra of SNARF-5F *in vitro* and inside human spermatozoa. Therefore, in order to convert the fluorescence emission values into the pH_*i*_, we performed an *in vivo* pH_*i*_ calibration with human and mouse spermatozoa using a high K^+^ solution combined with nigericin in order to equal pH_*i*_ to the pH_e_. This protocol is based on the assumption that the cytoplasmic K^+^ concentration is 120 mM in human and mouse spermatozoa, as determined in bovine spermatozoa (Babcock, 1983). Therefore, depending on the real cytoplasmic K^+^ concentration in human and mouse spermatozoa, the absolute pH_*i*_ values could be different. In our conditions, we determined that the pH_*i*_ value in non-capacitated human and mouse spermatozoa is 6.72 ± 0.19 and 6.63 ± 0.23, respectively. These values were measured in the head, but no significant differences were observed in the flagellum (see below). There are several reports of pH_*i*_ determinations (most of them in cell population experiments and a few using single cell determination) of non-capacitated spermatozoa from distinct mammalian species: 6.24 (Parrish et al., 1989a) and 6.7 (Vredenburgh-Wilberg and Parrish, 1995) in bovine spermatozoa, 6.55 (Balderas et al., 2013), 6.54 (Zeng et al., 1996), and 6.8 (Carlson et al., 2007) in mouse sperm, and 6.7 (Hamamah et al., 1996; Fraire-Zamora and González-Martínez, 2004) and 6.94 (Cross and Razy-Faulkner, 1997) in human spermatozoa. Our findings that pH_*i*_ values of non-capacitated mammalian spermatozoa are >6.5 are consistent with the

report that detergent-demembranated bovine spermatozoa do not exhibit motility at pH 6.5, although they are highly motile at pH 7.0 (Ho et al., 2002).

### Regional pH_*i*_ Difference in the Head and the Flagellum

We did not observe significant differences between the head and the flagellum in the basal pH_*i*_ in non-capacitated spermatozoa, although the head pH_*i*_ tends to be slightly higher than the flagellar pH_*i*_ both in human (6.72 ± 0.19 and 6.69 ± 0.24, respectively) and mouse spermatozoa (6.63 ± 0.23 and 6.60 ± 0.26, respectively). Our results are similar to those reported in bovine spermatozoa (Vredenburgh-Wilberg and Parrish, 1995). In general, the epifluorescent signal from an indicator incorporated into the sperm head is generally much higher than in the flagellum, independently of the species. Therefore, we examined if TIRF microscopy would reduce the fluorescence difference between the head and the flagellum. However, we did not observe significant differences nor advantages of TIRF microscopy compared to epifluorescence microscopy (Figure 4) either in mouse or human spermatozoa for measuring pH_*i*_. With these data, we can conclude that epifluorescence microscopy with SNARF-5F AM allows performing reliable single spermatozoa pH_*i*_ imaging with a good S/N ratio in both spermatozoa head and flagellum (mid piece of flagellum in the case of mouse).

### Difference of pH_*i*_ Responses to ${\text{HCO}}_{3}^{-}$ Between Human and Mouse Spermatozoa

${\text{HCO}}_{3}^{-}$ is an essential ion for mammalian sperm to acquire the ability to fertilize the oocyte (Lee and Storey, 1986). In fact, the ${\text{HCO}}_{3}^{-}$ concentrations in rabbit uterine and tubal fluids are approximately twice as high as in the blood plasma, which results in pH values of 7.4 and 8.1–8.3, respectively (Vishwakarma, 1962). In rhesus monkeys, the pH and ${\text{HCO}}_{3}^{-}$ concentration in the oviduct lumen change during the menstrual cycle. Namely, these values are similar to those of the blood plasma during the follicular phase, but they suddenly increase concomitantly with ovulation (Maas et al., 1977). This observation supports the importance of ${\text{HCO}}_{3}^{-}$ for fertilization in mammals. The principal role of cytoplasmic ${\text{HCO}}_{3}^{-}$ in mammalian spermatozoa is considered to be the activation of the soluble adenylyl cyclase, which increases cAMP (Okamura et al., 1985; Buck et al., 1999; Chen et al., 2000), leading to protein kinase A (PKA) stimulation. The enhanced PKA activity increases flagellar beat frequency (Wennemuth, 2003) and elevates CatSper activity (Carlson et al., 2003; Orta et al., 2018), among many other things.

In this work, we observed that ${\text{HCO}}_{3}^{-}$ elevates the pH_*i*_ in both human and mouse spermatozoa (Figure 7D). In contrast, Carlson et al. (2007) reported that ${\text{HCO}}_{3}^{-}$ did not induce a pH_*i*_ increase in mouse spermatozoa. Interestingly, we found a difference in the kinetics of ${\text{HCO}}_{3}^{-}$-induced pH_*i*_ increase between the two species (Figure 7D), namely a faster increase in mouse compared to human spermatozoa, but of similar magnitude (Figure 7C). So far, several mechanisms have been reported for ${\text{HCO}}_{3}^{-}$ influx, such as the NBC (Demarco et al., 2003), Cl^–^/${\text{HCO}}_{3}^{-}$ exchangers (Chavez et al., 2012), and the CFTR channel (Hernández-González et al., 2007; Xu et al., 2007). However, the physiological relevance of each transporter is unclear as well as differences between the two species. In addition to ${\text{HCO}}_{3}^{-}$ transporters, CO_2_ diffusion with subsequent hydration by intracellular CA contributes to an increase in cytoplasmic ${\text{HCO}}_{3}^{-}$ concentration (Carlson et al., 2007; Wandernoth et al., 2010; José et al., 2015). Curiously, a general CA inhibitor, ethoxyzolamide, potently affects human but not mouse sperm motility (José et al., 2015), suggesting a difference in the involvement of CAs in the motility of the two species. Another explanation is that human spermatozoa may have higher pH buffering capacity than mouse spermatozoa. This might be correlated to the time required for capacitation (>6 h in human compared to 1–2 h in mouse spermatozoa). Indeed, the pH_*i*_ of mammalian spermatozoa studied so far increases around 0.14–0.4 units during capacitation (Parrish et al., 1989b; Vredenburgh-Wilberg and Parrish, 1995; Hamamah et al., 1996; Zeng et al., 1996; Cross and Razy-Faulkner, 1997; Fraire-Zamora and González-Martínez, 2004; Balderas et al., 2013). A significant part of this pH_*i*_ change can be attributed to the ${\text{HCO}}_{3}^{-}$ influx into the cell. Therefore, further studies are required for a better understanding of the mechanism of ${\text{HCO}}_{3}^{-}$-induced pH_*i*_ increase during capacitation. The pH_*i*_ imaging system established in this study should contribute to this issue.

### Effect of Progesterone in Human Spermatozoa

Progesterone increases [Ca^2+^]_i_ in human spermatozoa at concentrations as low as 300 nM, through CatSper activation (Tesarik et al., 1992; Harper et al., 2003; Achikanu et al., 2018). Recently it was described that the progesterone receptor in these cells is a α/β hydrolase domain-containing protein (ABHD2), which depletes the endocannabinoid 2-arachinoylglycerol (2AG) from membrane and removes CatSper inactivation (Miller et al., 2016; Mannowetz et al., 2017). In contrast, there is inconsistency regarding how progesterone affects pH_*i*_. Some groups suggest that this hormone acidifies, others that it alkalizes or does not induce pH_*i*_ changes (Garcia and Meizel, 1996; Hamamah et al., 1996; Cross and Razy-Faulkner, 1997; Fraire-Zamora and González-Martínez, 2004). In the present work, progesterone did change pH_*i*_ in human spermatozoa even at concentrations as high as 10 μM (Figure 8). Our result supports that progesterone activates CatSper in a pH-independent manner, possibly exclusively via ABHD2-2AG.

Progesterone-induced Ca^2+^ influx through CatSper may affect the activity of transporters and enzymes that can affect pH_*i*_ such as PMCA (Wennemuth et al., 2003; Okunade et al., 2004) and NOX5 (Baker and Aitken, 2004; Musset et al., 2012). Both PMCA and NOX5 may acidify pH_*i*_ when they are activated, namely when the [Ca^2+^]_i_ is high. However, the pH_*i*_ acidification together with the membrane potential depolarization caused by Ca^2+^ influx through CatSper and electron efflux through NOX5 could activate Hv1 channel (Lishko et al., 2010; Berger et al., 2017) and may rapidly neutralize the acidification (alkalize the pH_*i*_). Depending on the experimental conditions, one activity (acidifying or alkalizing) may exceed the other when progesterone stimulates human CatSper. This may account for part of the discrepancies regarding human sperm pH_*i*_ responses to progesterone. Further studies are required to confirm this hypothesis.

## Data Availability Statement

All datasets generated for this study are included in the article/Supplementary Material.

## Ethics Statement

The studies involving human participants/donors were reviewed and approved by the Bioethics Committee of the Instituto de Biotecnología. The donors provided their written informed consent to participate in this study. The animal study was reviewed and approved by the Bioethics Committee of the Instituto de Biotecnología.

## Author Contributions

TN conceived the project. JC performed most of the experiments and prepared the figures. All authors proposed the experiments, discussed the results, and wrote, revised, and approved the manuscript.

## Conflict of Interest

The authors declare that the research was conducted in the absence of any commercial or financial relationships that could be construed as a potential conflict of interest.

## Supplementary Material

FIGURE S1 Spectral characterization of the pH sensitive dye SNARF-5F. Representative emission spectra in 405, 440, 465, 488, 510, 532, and 543 nm excitation wavelengths, in 50 mM potassium phosphate buffers at the proportions indicated in Table 1, obtaining pH_e_: 5.5, 6.0, 6.4, 6.8, 7.0, 7.2, 7.4, 7.8, and 8.2. The lines are representative fluorescence spectra at indicated excitation wavelength in each pH_e_; *n* = 3.

FIGURE S2 Single cell pH_*i*_ measurements with BCECF using laser of led excitation causes significant photobleaching and a reduced response to alkalization and acidification. Representative normalized recordings using BCECF-loaded spermatozoa in human **(A,B)** and mouse **(C,D)**. Laser **(A,C)** and LED **(B,D)** were used as the excitation light source. Arrows indicate the manual addition of 10 mM NH_4_Cl and 5 mM HCl in each panel. Each trace represents the response of a single cell; *n* = 3.
