# Supplementary figures and images for: Quantitative Intracellular pH Determinations in Single Live Mammalian Spermatozoa Using the Ratiometric Dye SNARF-5F
Source: Front Cell Dev Biol. 2020 Jan 17;7:366. doi: 10.3389/fcell.2019.00366 (PMC6978660; doi:10.3389/fcell.2019.00366)

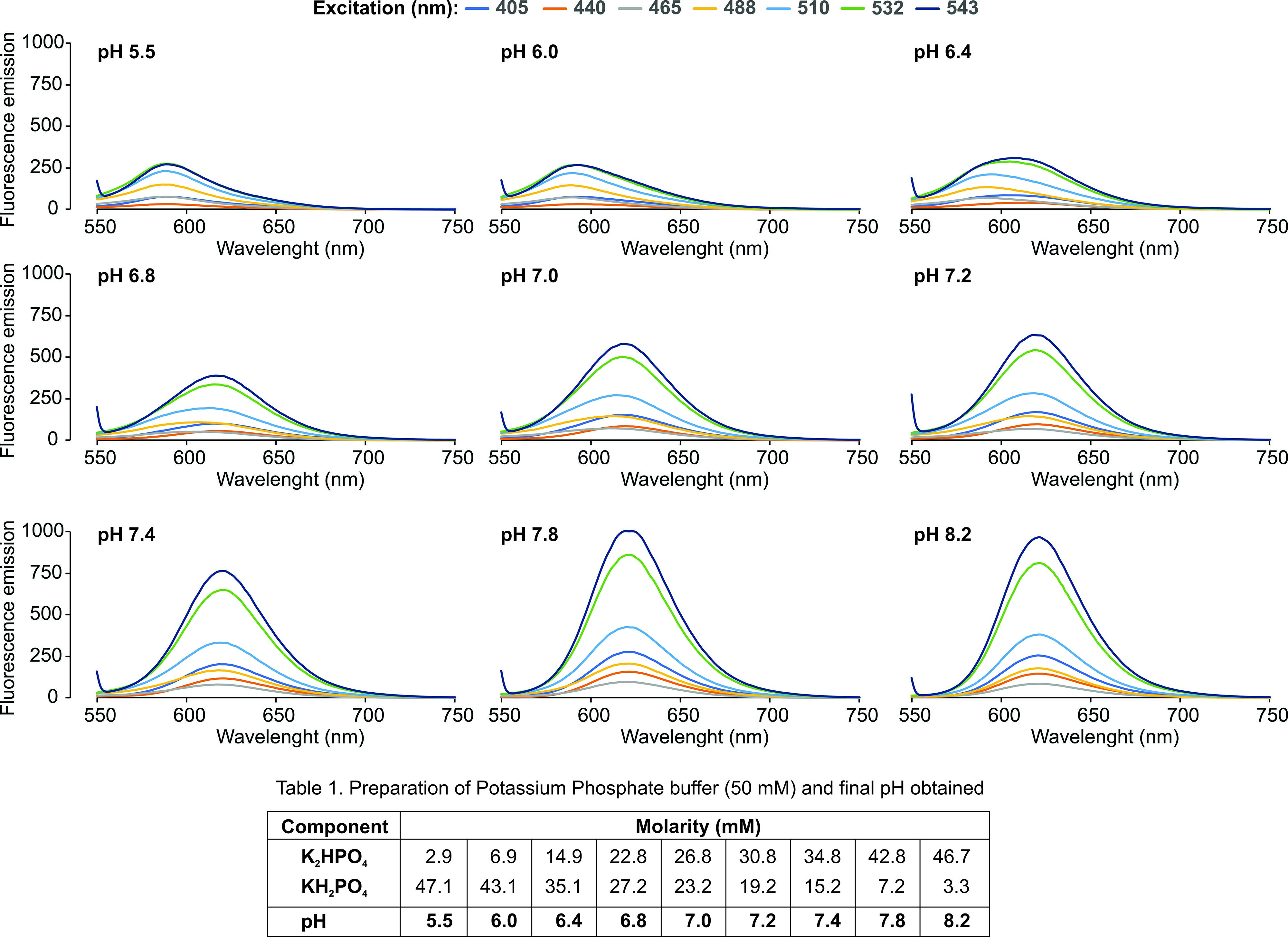

Supplement: FIGURE S1 — Spectral characterization of the pH sensitive dye SNARF-5F. Representative emission spectra in 405, 440, 465, 488, 510, 532, and 543 nm excitation wavelengths, in 50 mM potassium phosphate buffers at the proportions indicated in Table 1, obtaining pHe: 5.5, 6.0, 6.4, 6.8, 7.0, 7.2, 7.4, 7.8, and 8.2. The lines are representative fluorescence spectra at indicated excitation wavelength in each pHe; n = 3. [file Image_1.JPEG]

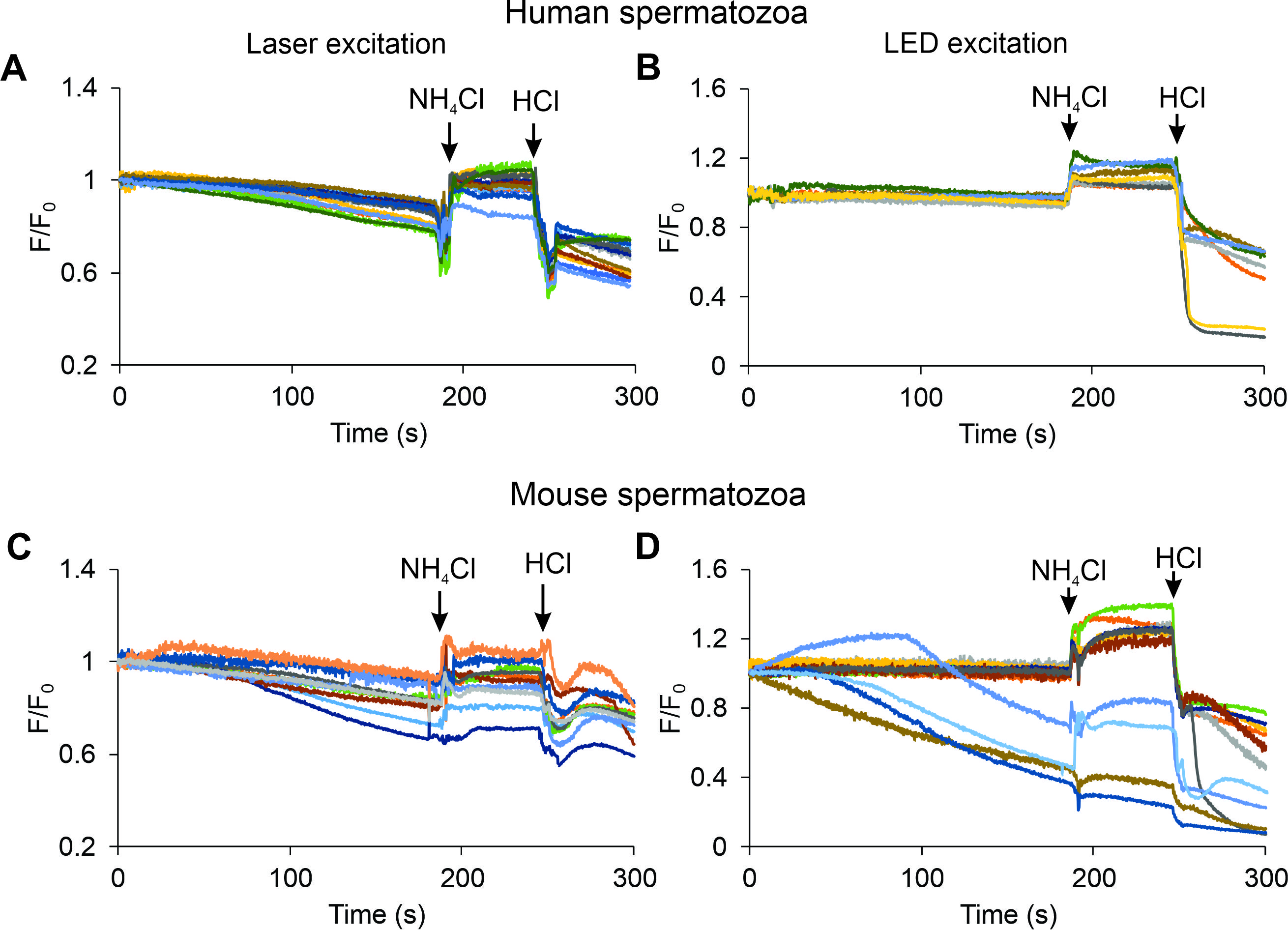

Supplement: FIGURE S2 — Single cell pHi measurements with BCECF using laser of led excitation causes significant photobleaching and a reduced response to alkalization and acidification. Representative normalized recordings using BCECF-loaded spermatozoa in human (A,B) and mouse (C,D). Laser (A,C) and LED (B,D) were used as the excitation light source. Arrows indicate the manual addition of 10 mM NH4Cl and 5 mM HCl in each panel. Each trace represents the response of a single cell; n = 3. [file Image_2.JPEG]
